# The Preventive Effects of Platelet-Rich Plasma Against Knee Osteoarthritis Progression in Rats

**DOI:** 10.7759/cureus.48825

**Published:** 2023-11-15

**Authors:** Haruka Takemura, Takayuki Okumo, Tokito Tatsuo, Kanako Izukashi, Hideshi Ikemoto, Naoki Adachi, Midori Mochizuki, Koji Kanzaki, Masataka Sunagawa

**Affiliations:** 1 Department of Physiology, Showa University Graduate School of Medicine, Tokyo, JPN; 2 Department of Orthopedic Surgery, Showa University Fujigaoka Hospital, Yokohama, JPN; 3 Department of Pharmacology, Showa University Graduate School of Medicine, Tokyo, JPN; 4 Department of Orthopedics, Showa University Fujigaoka Hospital, Yokohama, JPN

**Keywords:** osteoarthritis/koa, rotarod test, destabilization of the medial meniscus, rat model, platelet-rich plasma (prp)

## Abstract

Background: In recent years, the intra-articular administration of platelet-rich plasma (PRP), a novel therapeutic strategy for knee osteoarthritis (KOA), has gained attention. However, the efficacy of PRP in inhibiting degenerative joint changes remains unclear. The current study aimed to evaluate the therapeutic effect of the intra-articular administration of PRP in rats with induced KOA.

Materials and methods: PRP was prepared from the whole blood of nine-week-old male Wistar rats via centrifugation at 25°C, 200 × g, for seven minutes. KOA was induced in the right knees of the rats via destabilization of the medial meniscus (DMM) surgery. The animals were divided into the control, sham, DMM, and DMM + PRP groups (n = 5 each). The rats in the DMM + PRP group received 50 μL of intra-articular PRP in the right knee joint four weeks after surgery. The rotarod test was conducted to assess locomotive function. Eight weeks after DMM surgery, the degree of medial meniscus extrusion was measured via computed tomography (CT) images on the right knee. Then, a histological analysis of the harvested knees was conducted. KOA progression was assessed using the Osteoarthritis Research Society International (OARSI) score. The number of multinucleated tartrate-resistant acid phosphatase (TRAP)-positive osteoclasts in the subchondral bone was counted via histological analysis.

Results: The degree of medial meniscus extrusion did not significantly differ between the DMM and DMM + PRP groups. Similarly, there were no significant differences in the walking time based on the rotarod test between the DMM and DMM + PRP groups. However, the DMM group had a significantly higher OARSI score than the DMM + PRP group. The number of TRAP-positive osteoclasts in the subchondral bone of the DMM group increased over time, peaking four weeks after surgery. The DMM + PRP group had a higher number of TRAP-positive osteoclasts in the subchondral bone than the control group. However, there was no significant difference between the number of TRAP-positive osteoclasts between the DMM group and the control and sham groups.

Conclusion: The intra-articular administration of PRP may inhibit KOA progression in a rat model, especially in the articular cartilage degradation and osteophyte formation. The results can provide further evidence about the efficacy of PRP against KOA progression and can contribute to the current practice of healthcare professionals based on accurate knowledge.

## Introduction

Knee osteoarthritis (KOA) is one of the most common chronic joint diseases causing deformity in the articular cartilage, subchondral bone, and periarticular soft tissue, which impairs the quality of life (QOL) of the patients [1]. KOA is more common in people aged over 40 years, with approximately 300 million patients presenting with KOA worldwide. The number of patients with this condition is still increasing in most developed countries [2]. Nonsteroidal anti-inflammatory drugs and corticosteroids are recommended as nonsurgical treatments for relieving pain or swelling. However, the use of these drugs cannot inhibit KOA progression.

In cases in which QOL impairment cannot be improved by exercise and orthotics, surgical treatment such as total knee arthroplasty (TKA) or osteotomy around the knee may be required. Hence, the number of TKA can increase [3]. TKA is associated with financial burden in several countries. The medical cost of osteoarthritis in various countries ranges from 1% to 5% of the gross domestic product of these countries, with hip and knee joint replacements accounting for the major proportion of these healthcare costs [4]. Therefore, there is a need to develop effective and preventive conservative therapeutic options.

Recently, the intra-articular administration of platelet-rich plasma (PRP) has gained attention as a novel conservative treatment strategy for KOA. PRP is an autologous plasma concentrate prepared via the centrifugation of whole blood. The bottom layer of the plasma component formed after centrifugation, which is called the buffy coat, contains concentrated platelets (Plt) and white blood cells (WBC). PRP contains over 800 soluble proteins and molecules, and several growth factors released from platelets can promote angiogenesis and mesenchymal stem cell proliferation, which contribute to accelerated tissue healing [5]. PRP can contain high levels of interleukin (IL) 1 receptor antagonists and IL-4, which are important in modulating inflammatory responses [6]. The modulation of inflammatory mediators may shed light on treatment strategies for patients with osteoarthritis [7]. PRP was used for regenerative medicine, including ophthalmology, dermatology, and dentistry [8-10]. In the field of orthopedics, PRP has been used as a conservative treatment for KOA and as an adjunctive treatment for tendinopathy or nonunion [11,12]. PRP is mainly administered intra-articularly for treating KOA. In patients with KOA, PRP is reported to provide better clinical outcome than hyaluronic acid [13]. Some reports have shown that the intra-articular administration of PRP to patients with early KOA has improved cartilage lesions in the patellofemoral joint evaluated on MRI T2 mapping images [14].

However, the effect of PRP therapy on inhibiting degenerative changes in the affected joints remains unclear. The present study aimed to explore the therapeutic effect of PRP on a KOA rat model.

## Materials and methods

The Ethics Committee for Animal Experiments at the Showa University Animal Laboratory approved this study (approval number: 05079). This study used nine-week-old male Wistar rats (300-350 g). The animals were kept in cages of 3-4 rats per cage under the following conditions: room temperature of 24℃-26℃, humidity of 40%-50%, and a light/dark cycle of 12 hours/12 hours. Powdered rodent chow (CE-2, CLEA Japan, Tokyo, Japan) and water were provided ad libitum.

PRP was prepared via centrifugation of the rats’ whole blood, and a good PRP was defined as low hemoglobin (Hb) levels and high platelet (Plt) count [15,16]. To prepare PRP, 8 mL of whole blood was aspirated from the inferior vena cava or common iliac vein from five nine-week-old male Wistar rats under general anesthesia with isoflurane inhalation. Whole blood was then centrifuged at 25°C, 200 × g for seven minutes. A 10th of the whole blood, 800 μL of plasma concentrate with the viscous white layer called buffy coat between the plasma and blood cell, was collected and stored as PRP. PRP was frozen at -20°C until intra-articular injection and brought to room temperature before intra-articular administration. The white blood cell (WBC) count, Hb level, and Plt count were measured, and the differences in blood cell counts between the whole blood and PRP were compared in order to examine and confirm that prepared PRP was applicable for this experiment.

The destabilization of the medial meniscus (DMM) model [17] was applied to the KOA-induced rat model, which shows similar pathogenesis of KOA in humans [18,19]. DMM surgery was described in a previous report [20]. Briefly, under general anesthesia with isoflurane inhalation, a longitudinal incision was made in the right knee joint, and the medial joint capsule was dissected to expose the medial menisco-tibial ligament (MMTL). Transecting the MMTL can cause the loss of the stability of the medial meniscus [19]. The medial capsule and skin were sutured with 6-0 Vicryl (Ethicon, Inc., Somerville, NJ).

**Figure 1:**
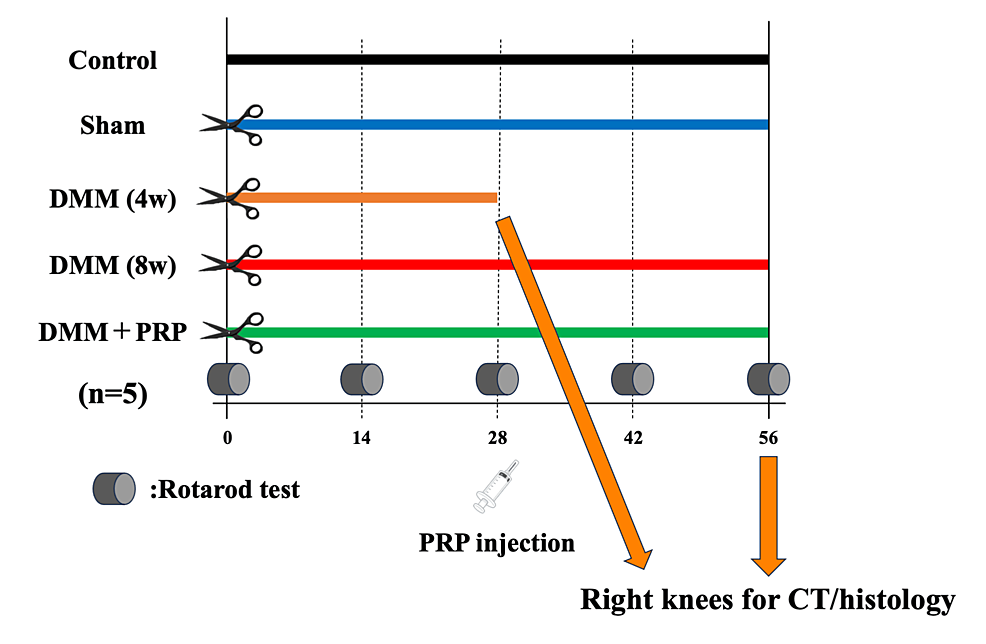
Experimental protocol. Nine-week-old male Wistar rats were divided into the control, sham, DMM (4W), DMM (8W), and DMM + PRP groups (n = 5 in each group). DMM surgery was performed on the right knee under general inhalation anesthesia. The DMM + PRP group received 50 μL of PRP in the right knee joint at 28 days after the surgery. The rotarod test was performed every two weeks and then at 56 days after surgery, a CT scan was performed, and the right knees were harvested for histological analysis. CT, computed tomography; DMM, destabilization of the medial meniscus; PRP, platelet-rich plasma

The animals were divided into the control, sham, DMM (4W), DMM (8W), and DMM + PRP groups (n = 5 each). The DMM + PRP group received 50 µL of PRP in the right knee joint four weeks after DMM surgery. Eight weeks postoperatively, the right side of the knee joints was harvested, except for the DMM (4W) group in which the knee joints were harvested four weeks after surgery (Figure 1). For eight weeks, the rotarod test was performed weekly to assess mobility and pain in rodents [21]. Briefly, the rats were set on an accelerating rotarod apparatus (LE8305, Panlab Harvard Apparatus, Barcelona, Spain) with a diameter of 60 mm and a lane width of 75 mm, which gradually accelerates. Then, the latency to fall off is measured. In the musculoskeletal disease model such as neuropathy and osteoarthritis, if the latency to fall was longer, the locomotor function was higher. In this study, the rotarod test was performed prior to and at 14, 28, 42, and 56 days after DMM surgery. The rats were acclimated to the rotarod apparatus for two days before the start of the experiment at 4 rpm for 180 seconds [21]. On each measurement day, the average of the three trials was calculated followed by confirming acclimatization to the measurement device.

**Figure 2:**
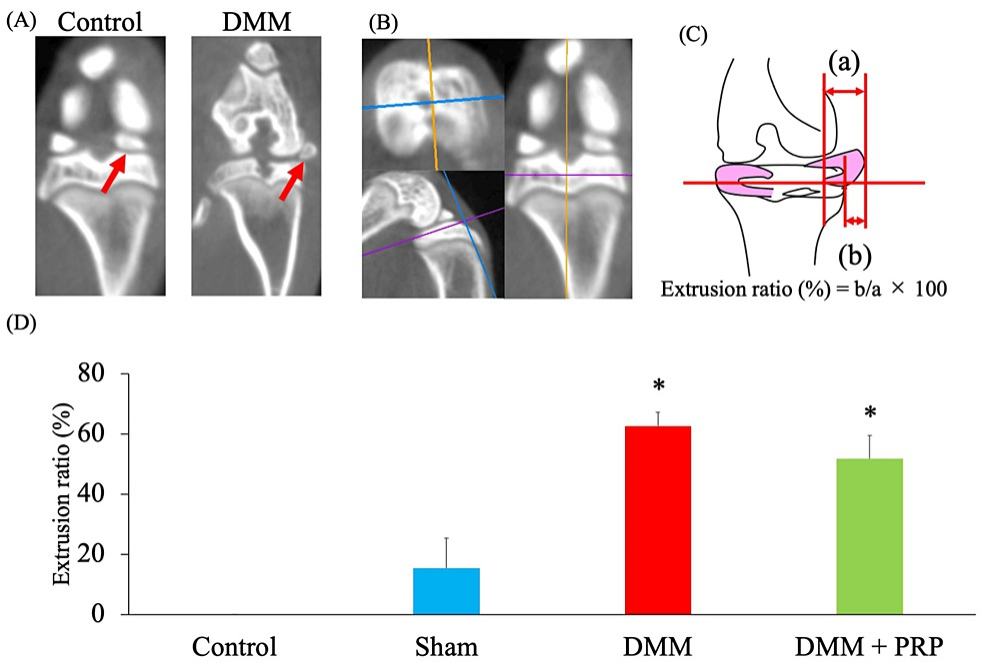
CT scan-based analysis of the medial meniscus extrusion ratio. (A) Representative CT scan of the right knee in the control and DMM groups. The medial meniscus (red arrow) is located above the medial tibial plateau, which is laterally extruded in the DMM group. (B) A coronal image was obtained. Then, the axial and sagittal referential lines were set. The medial meniscus extrusion rate at the view where the medial meniscus appeared to be the largest was evaluated. (C) The total transverse length of the medial meniscus and the length of the meniscus deviating from the articular surface, as shown in (a) and (b), respectively. MMER was defined and calculated as the ratio of the length of (b) to the length of (a), which was shown as a percentage. (D) MMER. The DMM and DMM + PRP groups had a significantly higher MMER than the control group; however, the two groups did not have a significant difference. *P < 0.05 versus the control group based on the ANOVA followed by the Tukey test. CT, computed tomography; DMM, destabilization of the medial meniscus; PRP, platelet-rich plasma; MMER, medial meniscus extrusion ratio; ANOVA, analysis of variance

Meniscus extrusion induced by DMM surgery was validated using a computed tomography (CT) scan image (Figure 2A). The medial meniscus extrusion ratio (MMER) was evaluated in a coronal section view after establishing axial and sagittal reference lines in which the medial meniscus was the largest (Figure 2B). MMER was defined and calculated as the ratio of the length of the medial meniscus that deviated from the edge of the tibial plateau to the total transverse length of the medial meniscus, which was shown as a percentage (Figure 2C). It can be interpreted that the higher the MMER, the more extraverted the medial meniscus. The DMM surgery makes the medial meniscus significantly instable, so the MMER is thought to increase.

For the histological analysis, rats were anesthetized with intraperitoneal sodium pentobarbital (50 mg/kg; Somnopentyl, Kyoritsu Seiyaku, Tokyo, Japan) and then perfused with phosphate-buffered saline at pH 7.4. The knee joint was fixed via perfusion fixation with 4% paraformaldehyde. Knee joint slices for tissue staining were prepared according to the Osteoarthritis Research Society International (OARSI) recommendations [22]. Briefly, samples were fixed in 4% paraformaldehyde for three days and then demineralized in ethylenediaminetetraacetic acid disodium for three weeks. These samples were embedded in paraffin. Next, 4 µm of the slices were prepared using Retratome (REM-700, Yamato Kohki Industrial Co., Ltd., Saitama, Japan). Slices were made every 200 µm anteriorly or posteriorly and were affixed to glass slides. Samples adhering to the glass slides were stained with toluidine blue and viewed using the BX53 microscope (Olympus, Tokyo, Japan). The OARSI score for rats [22] is composed of cartilage degeneration (0-15 points), subchondral bone destruction (0-5 points), and osteophyte formation (0-4 points). Therefore, the total score ranged from 0 to 24. A lower score indicated less joint degeneration. In the early stage of KOA, subchondral bone turnover deteriorates [23]. The number of tartrate-resistant acid phosphatase (TRAP)-positive osteoclasts in the subchondral bone was counted using the TRAP staining kit (Fujifilm Wako Pure Chemical Corporation, Osaka, Japan). TRAP-positive osteoclasts can be identified as multinucleated giant cells [24], and the number of these cells was measured. Data were expressed as mean ± standard deviation of multiple repetitions of the same experiment.

Statistical analyses were performed with the JMP® Pro version 16.0 software (SAS Institute Inc., Cary, NC), according to the Shapiro-Wilk normality test, and blood counts were performed with Student’s t-test. The rotarod test score, MMER, OARSI score, and the number of TRAP-positive osteoclasts were evaluated using a one-way analysis of variance and the Tukey test. Student’s t-test was used to evaluate the three OARSI sub-scores. P < 0.05 was considered statistically significant.

## Results

MMERs on CT scan images were 0%, 15.4 ± 10.0%, 62.6 ± 4.6%, and 51.7 ± 7.7% in the control, sham, DMM, and DMM + PRP groups, respectively. The DMM and DMM + PRP groups had significantly higher MMERs than the control group (P < 0.001 and P < 0.001, respectively). Thus, MMER did not significantly differ between the two groups (Figure 2D).

**Figure 3:**
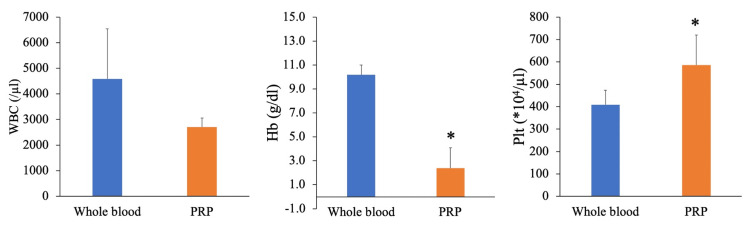
Blood cell count in the whole blood and PRP. In total, 8 mL of whole blood was aspirated from five nine-week-old Wistar rats, and PRP was prepared via centrifugation at 200 × g, for seven minutes. *P < 0.05 versus the whole blood using Student’s t-test. WBC, white blood cell; Hb, hemoglobin; Plt, platelet; PRP, platelet-rich plasma

The WBC counts were 4,580 ± 1,961/µL in the whole blood and 2,700 ± 360/µL in PRP. Hence, the results did not significantly differ. The Hb levels were 10.2 ± 0.8 g/dL in the whole blood and 2.4 ± 1.7 g/dL in PRP. Hence, PRP had a significantly lower Hb level than the whole blood. PRP had a significantly higher Plt count (5,860,000 ± 1,334,000/µL) than the whole blood (4,080,000 ± 661,000/µL) (Figure 3).

**Figure 4:**
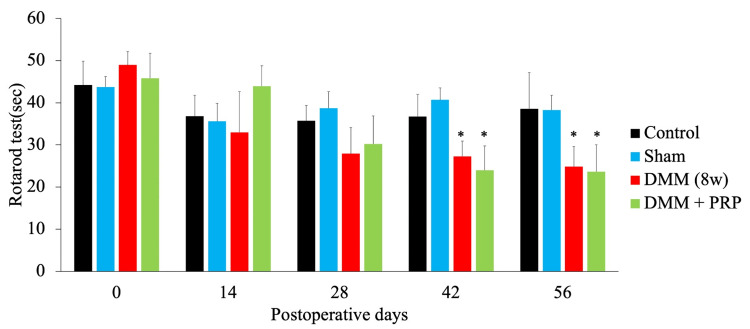
Rotarod test. The rotarod test was performed prior to or 14, 28, 42, and 56 days after DMM surgery. *P < 0.05 versus the control group based on the ANOVA followed by the Tukey test. DMM, destabilization of the medial meniscus; PRP, platelet-rich plasma; ANOVA, analysis of variance

In the rotarod test, there was no significant difference in walking time measured with the rotarod test before DMM surgery in all groups. The walking time of the DMM and the DMM + PRP groups significantly decreased at 42 (P = 0.010 and P = 0.001, respectively) and 56 (P = 0.007 and P = 0.013, respectively) days postoperatively compared with the control groups (Figure 4).

**Figure 5:**
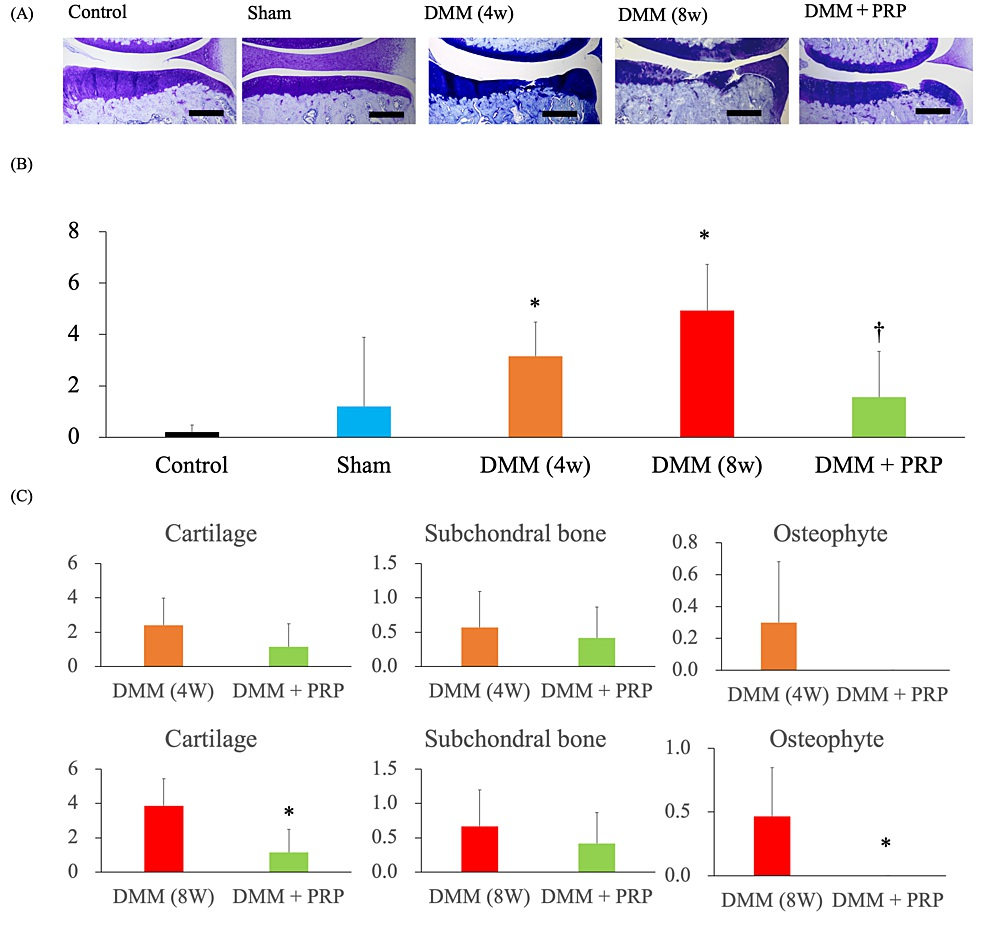
Histological analysis with OARSI score. (A) Representative views of the toluidine blue-stained right knee samples in each group. Magnification: ×40. Scale bars = 200 nm. (B) OARSI score. (*P < 0.05 versus the control group; ^†^P < 0.05 versus the DMM (8W) group based on the ANOVA followed by the Tukey test). (C) Comparison of OARSI sub-scores between the DMM and DMM + PRP groups (*P < 0.05 versus the DMM (8W) group based on Student’s t-test). DMM, destabilization of the medial meniscus; PRP, platelet-rich plasma; OARSI, Osteoarthritis Research Society International; ANOVA, analysis of variance

Toluidine blue staining of the rats’ paraffinized knee joint slices revealed articular cartilage degradation, subchondral bone destruction, and osteophyte formation in the DMM group (Figure 5A). The mean OARSI scores were 0.2 ± 0.3 in the control group, 1.2 ± 2.7 in the sham group, 3.2 ± 1.3 in the DMM (4W) group, 4.9 ± 1.8 in the DMM (8W) group, and 1.6 ± 1.8 in the DMM + PRP group. The DMM group had a significantly higher OARSI score than the control group (P = 0.005), and the DMM + PRP group had a significantly lower OARSI score than the DMM (8W) group (P = 0.048). However, the OARSI scores did not significantly differ between the DMM (4W) group and the DMM + PRP group (P = 0.614) (Figure 5B). Notably, the DMM + PRP group had significantly lower cartilage degeneration and osteophyte formation OARSI sub-scores than the DMM group (Figure 5C). However, the three subscales did not significantly differ between the DMM (4W) and the DMM + PRP groups.

**Figure 6:**
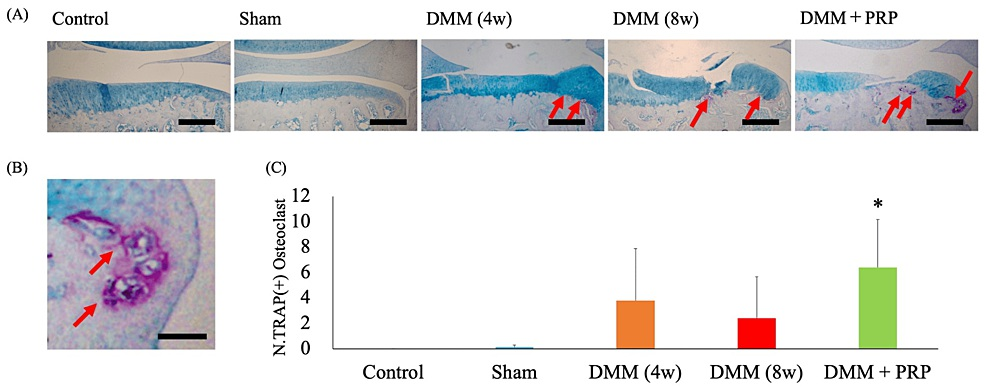
Multinucleated TRAP-positive osteoclast. (A) Representative views of TRAP staining of the right knee samples in each group. Magnification: ×40. Scale bars = 200 nm. (B) Multinucleated TRAP-positive osteoclast (red arrow). Magnification: ×400. Scale bars = 20 nm. (C) The number of TRAP-positive osteoclasts in the subchondral bone. *P < 0.05 versus the control group based on the ANOVA followed by the Tukey test. DMM, destabilization of the medial meniscus; PRP, platelet-rich plasma; TRAP, tartrate-resistant acid phosphatase; ANOVA, analysis of variance

TRAP staining in the medial tibial plateau revealed multinucleated TRAP-positive osteoclasts in the subchondral bone and osteophytes (Figure 6A, 6B). The numbers of osteoclasts were zero in the control group, 0.1 ± 0.2 in the sham group, 3.8 ± 4.1 in the DMM (4W) group, 2.4 ± 3.3 in the DMM (8W) group, and 6.4 ± 3.8 in the DMM + PRP group. The number of osteoclasts in the DMM + PRP group was significantly higher than that in the control group (P = 0.002). However, the number of osteoclasts did not significantly differ between the DMM (4W), DMM (8W), and DMM + PRP groups (P = 0.622 and P = 0.225, respectively) (Figure 6C).

## Discussion

In this study, the intra-articular injection of PRP revealed a preventive effect on KOA through the inhibition of the articular cartilage degeneration and osteophyte formation, even though it did not improve MMER, the morphological change via DMM surgery. Etiology for KOA was mainly divided into two factors, the mechanical factors [25,26] and the biological factors [15], so that the PRP treatment may have some influences on the biological properties in articular cartilage or synovium, which are associated with joint inflammation [14,15].

In recent years, there have been several reports about secondary KOA caused by the medial meniscus posterior root tear (MMPRT) [25]. This injury causes damage to the edge of the medial meniscus making the meniscus deviate outward from the medial tibial plateau. If the meniscus deviates, the load-bearing force will increase up to 200% [26]. If the mechanical force increases, articular cartilage and subchondral bone receive excessive physical stimulation, leading to KOA progression. In addition to “meniscus injury,” meniscus lesions have recently been shown to be frequently associated with an abnormal position, which is termed “meniscus extrusion.” Meniscus extrusion is defined as the meniscus being positioned extraarticular from the medial bony tip of the femur or tibia. Meniscus extrusion due to the disruption of the hoop structure of the meniscus following an anterior or posterior root meniscal injury increases the risk of developing and progressing osteoarthritis of the knee due to the inability to distribute mechanical loads to the area where the meniscus was originally located [27].

The pathophysiology of DMM in the rat model is similar to that of MMPRT; the meniscus significantly deviates after the surgery [19], and higher MMER indicates meniscus extrusion. In this study, there was no difference in the DMM and DMM + PRP groups in terms of MMER, suggesting that the administration of PRP in the DMM rat model did not improve MMER.

The rotarod test is used to investigate motor coordination and locomotive function in rodents [21]. The rotarod test scores of the rat models of musculoskeletal disease, such as those in this study, primarily reflect the pain and dysfunction associated with disease progression. The rotarod test score of the DMM rat model showed that motor function was already weakened four weeks after DMM surgery [19]. In addition, the administration of PRP four weeks after DMM surgery did not improve decreased motor function. On the other hand, PRP therapies may be a viable option in the DMM-induced post-traumatic osteoarthritis mouse model [28]. In the present study, PRP was administered four weeks after DMM surgery, and KOA progression was inhibited eight weeks postoperatively. As in a previous report, KOA develops four weeks postoperatively [20]. The administration of PRP may inhibit further KOA progression.

TRAP staining was performed on the knee joint eight weeks after DMM surgery to measure the number of TRAP-positive osteoclasts. It is reported that the bone remodeling rate in early KOA increases up to 20 times faster than those in the normal bone, and the bone remodeling biological factors increase [29]. In this study, similar changes were observed after DMM surgery such as the proliferation of TRAP-positive osteoclasts and osteophyte formation. The DMM + PRP group had significantly more osteoclasts than the control group, even though there was no significant difference in terms of osteoclast counts between the DMM and DMM + PRP groups.

The current study had several limitations. First, even though the pathological changes observed with the DMM rat model are similar to that of human KOA, this does not mean that these changes can be directly applicable to humans. Because of the underlying difference in locomotion between humans and rats, orthogonal bipedal and quadrupedal walking, the magnitude of the contribution of the meniscus to stabilizing joint kinematics is expected to be somewhat different. Second, the appropriate timing of PRP administration remains unclear. PRP was administered four weeks after DMM surgery. However, its therapeutic effect when PRP is administered before KOA develops needs to be investigated. Clinically, PRP is reported to be more effective when administered at the very early stages of KOA in humans than at the final stages of KOA. Therefore, performing an experiment with an early administration of PRP would be considered, i.e., one or two weeks after DMM surgery. Third, PRP therapy in humans significantly improved clinical outcomes compared to hyaluronic acid and corticosteroids 6-12 months after treatment [30]. Therefore, it is necessary to investigate the long-term effects of intra-articular PRP injection in the future. Finally, in the present study, PRP was prepared at 200 × g and had a higher platelet content than whole blood. However, comparative studies with other preparation protocols must be performed to determine the optimal preparation protocol.

## Conclusions

In this study, the intra-articular administration of PRP could inhibit KOA progression in the rat model, especially in the articular cartilage degradation and osteophyte formation. The results may at least provide further evidence about the efficacy of PRP against KOA for healthcare professionals with experimental elucidation. It can be used in future studies that aim to improve the management and outcomes of patients with KOA. Nevertheless, further studies should be needed to investigate the appropriate timing for intra-articular injection and the long-term effect of PRP.
